# Early Life Stress Impairs VTA Coordination of BLA Network and Behavioral States

**DOI:** 10.1523/JNEUROSCI.0088-24.2025

**Published:** 2025-02-13

**Authors:** Bradly T. Stone, Pantelis Antonoudiou, Eric Teboul, Garrett Scarpa, Grant Weiss, Jamie L. Maguire

**Affiliations:** Department of Neuroscience, Tufts University School of Medicine, Boston, Massachusetts 02111

**Keywords:** basolateral amygdala, dopamine, early life stress, oscillations, ventral tegmental area

## Abstract

Motivated behaviors, such as social interactions, are governed by the interplay between mesocorticolimbic structures, such as the ventral tegmental area (VTA), basolateral amygdala (BLA), and medial prefrontal cortex (mPFC). Adverse childhood experiences and early life stress (ELS) can impact these networks and behaviors, which is associated with increased risk for psychiatric illnesses. While it is known that the VTA projects to both the BLA and mPFC, the influence of these inputs on local network activity which govern behavioral states—and whether ELS impacts VTA-mediated network communication—remains unknown. Our study demonstrates that VTA inputs influence BLA oscillations and entrainment of mPFC activity in mice and that ELS weakens the ability of the VTA to coordinate BLA network states, while also impairing dopaminergic signaling between VTA and BLA. Optogenetic stimulation of VTA_BLA_ terminals decreased social interaction in ELS mice, which can be recapitulated in control mice by inhibiting VTA→BLA communication. These data suggest that ELS impacts social reward via the VTA→BLA dopamine network.

## Significance Statement

It is well established that oscillatory states in the basolateral amygdala (BLA) govern behavioral states. However, a gap in our knowledge exists regarding the mechanisms mediating transitions between BLA network states. Here we demonstrate a novel mechanism modulating BLA network states involving dopamine inputs from the VTA. Further, we demonstrate that early life stress, a major risk factor for psychiatric illnesses, impairs the ability of dopaminergic inputs from the VTA to coordinate BLA and mPFC network states. Thus, this study provides a novel mechanism mediating transitions between oscillatory states in the BLA which are well documented to govern behavioral states and demonstrates pathological perturbations in the ability of the VTA to coordinate BLA network states following early life stress.

## Introduction

Adverse childhood experiences (ACEs) are major risk factors for psychiatric illnesses. For example, maternal neglect is known to impair cognition, weaken resiliency toward future stressors, and increase the propensity to develop mood and substance use disorders in affected offspring (Bolton et al., 2018; Reshetnikov et al., 2020; for review see Delavari et al., 2016). Recent reports show that roughly 20% of children in the United States are exposed to ACEs and research has found a striking comorbidity between frequency and severity of ACEs with psychiatric illnesses (Humphreys et al., 2020; Sheridan and McLaughlin, 2020; Berman et al., 2022). In conjunction with these findings, decades of work have implicated the mesocorticolimbic system (MCL) as being a psychophysiological focal point due to its susceptibility to the effects of stressors (Sullivan and Dufresne, 2006; Butts et al., 2011), including early life stress (ELS; Rodrigues et al., 2011; Ventriglio et al., 2015), and its involvement in proper establishment and maintenance of reward processing and subsequent motived behaviors (Fibiger and Phillips, 1988). These epidemiological and deductive research studies support a role for the MCL in contributing to behavioral deficits associated with early life adversity; however, the mechanisms through which ELS influences the MCL to induce altered behavioral outcomes remains unclear. Revealing these mechanisms has the potential to provide both useful information regarding the pathophysiology of disease and potential novel avenues for therapeutic intervention.

Many aspects of motivation involve dopaminergic (DA) neurons within and across the MCL. Further, studies have shown that the MCL DA system is not fully developed until adulthood in both humans (Rothmond et al., 2012) and rodents (Voorn et al., 1988), making adolescents particularly vulnerable to ACEs and ELS, respectively (Bayer et al., 1993). Specifically, in rodents, the MCL DA system hits maturity on postnatal day 21 (P21; Voorn et al., 1988) with hindbrain features (e.g., neurons and neuropeptides) known to be structurally and functionally altered following ELS by this timepoint (Dubé et al., 2015; Bolton et al., 2018). While a growing body of research has narrowed the impact of ELS to a subset of nodes within the MCL, the mechanisms mediating the developmental impact of altered DA signaling on emotional processing and motivation are not fully understood.

Accumulating evidence from our lab and others demonstrates that network states (distinct oscillatory activity, quantified as local field potentials; LFPs) in the basolateral amygdala (BLA) govern behavioral states (Cao et al., 2016; Antonoudiou et al., 2022; Fu et al., 2022), particularly those involved in valence processing (Nukitram et al., 2021; DiLeo et al., 2022). Moreover, the BLA is well established as the primary emotional hub within the mammalian brain (Wolf et al., 2004; Namburi et al., 2016; Ju and Beyeler, 2021). Previous research has also shown that the VTA (Martínez-Hernández et al., 2006; Valencia-Torres et al., 2017) and connections between the VTA and regions across the MCL (Lowes et al., 2021; Smith and Torregrossa, 2021; Sun et al., 2021) are necessary for proper regulation of incentive attributions of salience to stimuli, which influence behaviors. The VTA has direct projections to the BLA (Floresco and Maric, 2007; Lüthi and Lüscher, 2014; Correia and Goosens, 2016), which monosynaptically projects to the medial prefrontal cortex (Floresco and Maric, 2007; Cheriyan et al., 2016; Sun et al., 2019; mPFC; anterior region of the MCL)—a region also known to be susceptible to stress (Shansky and Morrison, 2009; Belleau et al., 2019) and is involved in motivation processing (Somerville et al., 2013; Ko, 2017; Yang et al., 2022). Therefore, it is reasonable to hypothesize that VTA inputs into the BLA likely influence motivated behaviors.

Despite the acknowledgment of the VTA's projections to both the BLA and mPFC, the specific influence of these DA pathways on local network dynamics and motivated behaviors remains elusive. Moreover, the impact of ELS on VTA→BLA communication and the mechanisms mediating the subsequent behavioral consequences have yet to be fully elucidated. This study addresses these gaps in knowledge by examining the intricate modulatory role of the VTA in regulating oscillatory states in the BLA and, in turn, influencing mPFC activity and social motivation. In foundational experiments within control DAT-Cre animals, where Cre-dependent opsins were expressed in the VTA, we demonstrate that DA released from the VTA is sufficient to drive BLA activity across a range of stimulation frequencies. We go on to show that while the VTA robustly controls BLA and entrains mPFC network states, driving BLA→mPFC coherence, ELS diminishes these effects. Further, we show that activation of VTA projections to the BLA results in social avoidance in ELS mice but not in control mice with VTA→BLA DA signaling intact. Inhibition of these VTA efferents increases social avoidance in control mice, further supporting this network's involvement in stress vulnerability, and its control over stimulus incentive assignment. These findings have implications for our understanding of the etiology and potential therapeutic interventions for psychiatric disorders marked by disrupted motivation and reward processing.

## Materials and Methods

### Animals

C57BL/6J mice were housed at Tufts University School of Medicine in a temperature- and humidity-controlled environment, maintained on a 12 h light/dark cycle (lights on at 7 A.M.) with *ad libitum* access to food and water. All animal procedures were handled according to the protocols approved by the Tufts University Institutional Animal Care and Use Committee (IACUC).

### Early life stress paradigm

Pregnant C57BL/6J dams were purchased directly from Jackson Laboratory and were delivered and singly housed on day 16 of gestation (E16) at Tufts University School of Medicine. These cages were checked daily for litter births, at which time litters were randomly assigned to standard-rearing (control; CNT) or maternal separation (ELS) groups. ELS began on P1 and consisted of the removal and placement of the dam into a clean cage, out of the sight of her litter, for 3 h (beginning at 9 A.M.) for 5 d per week from P1 toP21. Offspring were weaned on P21. For all experimental conditions, ELS and CNT animals were age and litter matched.

### Stereotaxic surgery

Adult (>P60) male CNT and ELS mice were anesthetized by intraperitoneal injection (i.p.) of a ketamine/xylazine cocktail (90–120 mg/kg and 5–10 mg/kg, respectively). Before the onset of the procedure, sustained release buprenorphine (0.5–1 mg/kg) was administered subcutaneously as postoperative analgesia. The head of the mouse was shaved, antiseptic solution was applied, and an incision on the scalp was made to expose the skull. For viral injections, mice were unilaterally injected with a virus targeted at either the BLA or ventral tegmental area (VTA; see Table 1 for details) using a pulled glass pipette at a flow rate of 100 nl per minute using positive pressure from a 10 ml syringe. All viruses were purchased from Addgene. Post hoc imaging was performed to validate targeted viral expression.

**Table 1.** Stereotaxic viral injection methods

Figure	Virus	Titer (vg/ml)	Volume (nl)	Dilution	Region	From bregma (A/P, M/L, D/V)
Figure 1	AAV-DIO-ChrRimson-tdT	5 × 10^12^	300	NA	VTA	−3.10, −0.53, −4.50
Figures 2–7	AAV9-syn-ChrRimson-tdT; ChrR	1 × 10¹³	300	NA	VTA	−3.10, −0.53, −4.50
Figure 5	AAV9-hSyn-GRAB_DA2m_	1 × 10¹³	600	1:2	BLA	−1.35, −2.90, −4.70
Figure 5	AAV9-hSyn-eGFP	1 × 7^12^	300	NA	VTA	−3.10, −0.53, −4.50
Figure 7	AAV-hsyn-Jaws-KGC-GFP-ER2	1 × 10¹³	300	NA	VTA	−3.10, −0.53, −4.50

### LFP recordings

LFP recordings were performed in awake, adult C57BL/6J CNT and ELS mice, using custom-designed headmounts (Pinnacle 8201-C). These headmounts consisted of two depth electrodes (PFA-coated stainless steel wire, A-M Systems) targeted at the medial prefrontal cortex (mPFC; from bregma: AP: +1.75; ML: −0.30; DV: −2.0 mm) and BLA (from bregma: AP: −1.35; ML: −3.20; DV: −5.10 mm). The headmounts were affixed to the skull using stainless steel screws as ground and reference following 2.5 weeks from viral injection. The LFP data were bandpass filtered (1–250 Hz, Chebyshev Type II filter), and spectral analysis was performed in Python using custom-made scripts utilizing the fast Fourier transform. Specifically, spectral analysis of the LFP/EEG signal was performed using the short-time Fourier transform (STFT) with a 5 s Hann window and 50% overlap as done previously (Antonoudiou et al., 2022). The power line noise (59–61 Hz) was removed and values were filled using nearest interpolation. Outliers in each spectrogram were identified using a two-stage process. Firstly, a time-series was obtained from the mean power across frequencies of each spectrogram. Large deviations, defined as those greater than the mean plus 4 times the standard deviation, were replaced with the median of the nonoutlying data. Then, a sliding window method was applied to detect more subtle outliers based on local context, using 5× the median absolute deviation (MAD) of each 5 min window. Resulting outliers were removed and replaced with forward fill interpolation of the nearest values. All resulting time-series, obtained from the mean power across frequencies, were manually verified.

For optogenetic experiments, CNT and ELS mice expressing either ChrR or Jaws (Table 1) were implanted with an in-house modified version of the described headmounts, optrode, which consisted of the addition of an optic fiber (200 µm, 0.22 NA; Thorlabs) attached to the depth electrode aimed at BLA. Photostimulation was performed with a red light delivered through the optic fiber coupled to a red laser (640 nm, max power = 500 mW, Laserglow Technologies). The light intensity setting used for all optogenetic experiments in awake mice was measured at 30–40 mW from the end of the opto-cannula (Thorlabs; 200 µm, 0.39 NA). Sine waveforms for photostimulation were generated through LabChart (ADInstruments). The LFP data were bandpass filtered between 3 and 300 Hz.

For pulsed photostimulation experiments (5, 10, 20, and 40 Hz), stimulations were pseudorandomized, where each stimulation frequency was repeated three times, and post hoc analyses revealed no presentation order effect on power elevation. The average response was used for further quantification. The power spectral densities for optogenetic experiments were obtained in 1 s bins (50% overlap) using custom-made Python scripts. For LFP power ratio, quantification of the power of the oscillations was obtained at approximately ±1 Hz of each stimulation frequency during (0−120 s) and before stimulation (0−30 s). Only stimuli that produced phase-locked LFP responses ±2 standard deviations from group average were included. The continuous Morse wavelet transform (Lilly and Olhede, 2012) was used to visualize opto-spectrograms. For LFP coherence analyses, BLA→mPFC LFP synchronization was quantified using the phase locking value (PLV; Lachaux et al., 1999), obtained around bandpass-filtered ±1 Hz of each stimulation frequency, using custom-made Python scripts. These values were normalized across frequencies as a ratio between stimulation period and baseline period, as done for power-ratio analyses.

### Recording in vivo dopamine responses in BLA

Biosensing of dopamine binding was performed in awake, adult CNT and ELS animals, using the GRAB-DA sensor and the nVoke miniaturized microscope (an integrated imaging and optogenetics system, 455 nm blue GCaMP excitation LED, 590 nm amber optogenetic LED, Inscopix). Following injection of ChrR in VTA and GRAB_DA2m_ in BLA (Table 1), an Inscopix lens affixed to a baseplate (Inscopix, 1050-004413) was placed in the BLA. Mice were allowed to recover for 7 weeks prior to experimentation to allow for optimal viral expression and in vivo imaging. On the recording day, each animal was removed from their home cage, placed in a testing cage (a cage identical to home cage, containing fresh bedding/nesting), and had the miniature integrated microscope system (nVoke HD 2.0; Inscopix) attached to their baseplate. Each animal was given 10 min to habituate to the testing cage, at which time images were acquired using data acquisition software (ver. 2.0.0; Inscopix) at 20 frames per second, 20% of LED power, and a gain kept between (2 and 5, varying based on clarity of field of view; FOV). Images were acquired across a 10 min session consisting of 15 trials of 40 hz, 5 s pulse trains (5 ms width) of 620 nm LED (20 mW) light initiated every 30 s.

Acquired imaging data were downsampled (1/2 spatial binning), preprocessed, motion corrected, DA transients of ROIs (three concentric circles as detailed in Inscopix Processing Guidelines: https://iqlearning.inscopix.com/best-practices/applications/nt-imaging/single-color/processing) were extracted, and a change in fluorescence (Δ*F*/*F*) was computed using Inscopix Data Processing Software (ver. 1.9.2; Inscopix). All extracted traces were then subjected to a series of custom-built Python scripts which quadratically detrended the DA signals (to account for photobleaching across a session; application of a second-degree polynomial fit as seen in Zarahn et al., 1997; Taylor et al., 2021), normalized each ROI to the mean baseline (prestimulation) periods, and then parsed these signals into individual trials for analyses. Given that the DA response is intrinsically linked to the available DA in the region of recording, all statistical tests were computed using the trials as observations, thus accounting for the dependent nature of DA depletion across a session.

### Three-chamber social interaction task

We recorded behavior from adult CNT and ELS mice during a three-chamber social interaction task (3CST) following 3 weeks postviral infusion in a Plexiglas rectangular box (71 cm × 30 cm × 35 cm), made up of three identically shaped compartments, without a top. The compartments were separated by a transparent piece of Plexiglas, with cut-outs to allow for free movement between each compartment. The left and right compartments each had an inverted wire cup (diameter 8 cm) placed in the center of them, which had a designated depressed (1/4 inch lower) cylindrical section to ensure wire cups remained in place throughout testing. The apparatus and wire cups were thoroughly cleaned with 70% ethanol between each trial.

The 3CST consisted of two sessions (Toy-Toy and Toy-Female), each made up of a habituation and testing trial. The testing protocols were identical in structure made up of four trials, each with the inverted cups holding either a toy or a novel, female conspecific: mice explored the arena and toys (Toy-Toy session; habituation) for 10 min, they repeated this with the exception that one of the sides (testing; pseudorandomly chosen) triggered photostimulation upon physical entrance into the interaction zone (3 cm space directly extending beyond the wire cup), they begin a new habituation trial where they can explore and interact with a toy or a female mouse (Toy-Female; habituation), and finally they explore the arena with the same female mouse, triggering photostimulation upon entrance into the interaction zone around her wire cup (testing). During interaction zone triggered stimulation, mice received VTA_BLA_ 30–40 mW photostimulation through the optic fiber coupled to a red laser (640 nm) at 40 Hz (Table 2).

**Table 2.** 3CST sample protocol

Animal	Session	Trial	Stim.	Stim. Side	Detail
Animal 1	Toy-Toy	Habituation	Off	–	Toy inside cage on both sides of area
		Testing	On	L	Toy inside cage on both sides of area
	Toy-Female	Habituation	Off	–	Toy inside L cage, Female inside R cage
		Testing	On	R	Toy inside L cage, Female inside R cage
Animal 2	Toy-Female	Habituation	Off	–	Toy inside R cage, Female inside L cage
		Testing	On	L	Toy inside R cage, Female inside L cage
	Toy-Toy	Habituation	Off	–	Toy inside cage on both sides of area
		Testing	On	R	Toy inside cage on both sides of area

Stim., stimulation; R, right; L, left.

The overall activity of the test mouse in the 3CST was automatically recorded with the EthoVision XT video tracking system (Noldus). The amount of time that test mice spent in each chamber and the immediate vicinity (3 cm) of the cages (termed as “interaction zone”) was measured. A custom-built Arduino script was used to receive inputs from EthoVision XT, which detailed when an animal was in the interaction zone, causing the 640 nm laser to send the 40 hz pulse trains (described above) indefinitely, until the animal left the interaction zone. Social preference scores (PS) were calculated as the percentage of time spent in the stimulation side (*T*_S_) out of the total time spent in either left or right side of the area (*T*_S_ *+* *T*_NS_; Eq. 1). Normalized preference scores (NS) were calculated as the ratio between the preference score observed during the testing trial (PS_T_) and the preference score observed during the habituation trial (PS_H_; see Eq. 2). Here, time (*T*) is the cumulative seconds that the nose of the test mouse was detected in the interaction zone (which ensures actual interaction between test mouse and caged object; toy or female mouse). In this manner, we were able to unbiasedly quantify interaction preference, whereby a PS value >1 indicates more time spent interacting on the stimulation side. An NS value >1 indicates the 640 nm stimulation (testing trials) increased this interaction time, in comparison with nonstimulated (habituation) trials.

$$\mathrm{Preference}\;\mathrm{Score}\;(\mathrm{PS})\;=\;100\times \;\frac{T_{S}}{\left(T_{\mathrm{NS}}+\;T_{S}\right)}\;(1)$$

$$\mathrm{Normalized}\;\mathrm{Preference}\;\mathrm{Score}\;(\mathrm{NS})=100\times \;\frac{PS_{T}}{PS_{H}}.\;(2)$$

Female conspecifics in estrous (most receptive to mating) verified using vaginal lavage and microscopy according to Cora et al. (2015) were used as the social stimulus. Groups were counterbalanced for order of light stimulation as well as side assigned as the social zone (see Table 2 for details). A three-way mixed ANOVA (lifegroup × stimulation side × session) was performed and revealed no significant difference (*p* > 0.05), revealing that the session presentation order, nor side the stimulation occurred on, affected CNT and ELS animals differently.

### Quantification and statistical analysis

All statistical analyses were performed using Python (Python Software Foundation). Multigroup tests were tested with ANOVAs followed by post hoc multiple comparisons. A Greenhouse–Geisser correction was applied where appropriate and when assumption of sphericity was violated. For electrophysiological parameters between two groups, Mann–Whitney *U* tests were run with Benjamini/Hochberg procedure to control for false discovery rate (statsmodels). For comparison of ELS and CNT, appropriate one-way tests were applied, chosen based on distribution of data and test assumptions. The statistical package Pingouin was used for all tests, except for correlation analyses and patch-clamp electrophysiological comparisons. All tests and results are reported for each comparison, named in accordance with the figure the results detail. A *p* value <0.05 was considered statistically significant.

## Results

**Figure 1.**
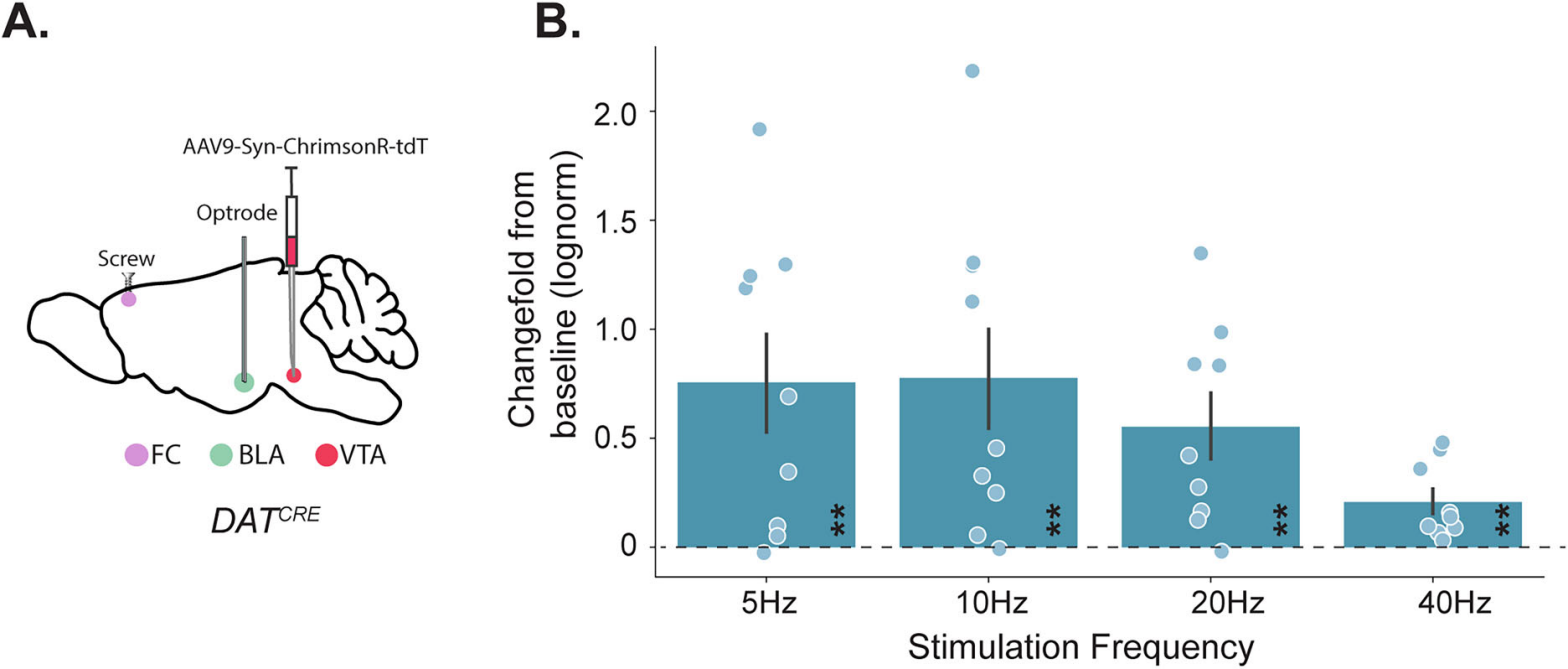
VTA_BLA_ photostimulation DAT-Cre mice drives network activity. inhibits ability for VTA_BLA_ to drive network activity. ***A***, Schematic of sagittal view shows at P70, in DAT-Cre mice (*N* = 7), AAV-Syn-ChrimsonR-tDT was injected into VTA, with an optrode placed into BLA while a grounding screw was placed over frontal cortex (FC). ***B***, Summary of LFP power ratio from BLA between pulsed stimulation and baseline periods (baseline = 0) where power was measured at each stimulation frequency for three, 2 min trials. Asterisks within bars indicate significant change from baseline. *p < 0.05, **p < 0.01, ***p < 0.001. Error bars represent SEM.

**Figure 2.**
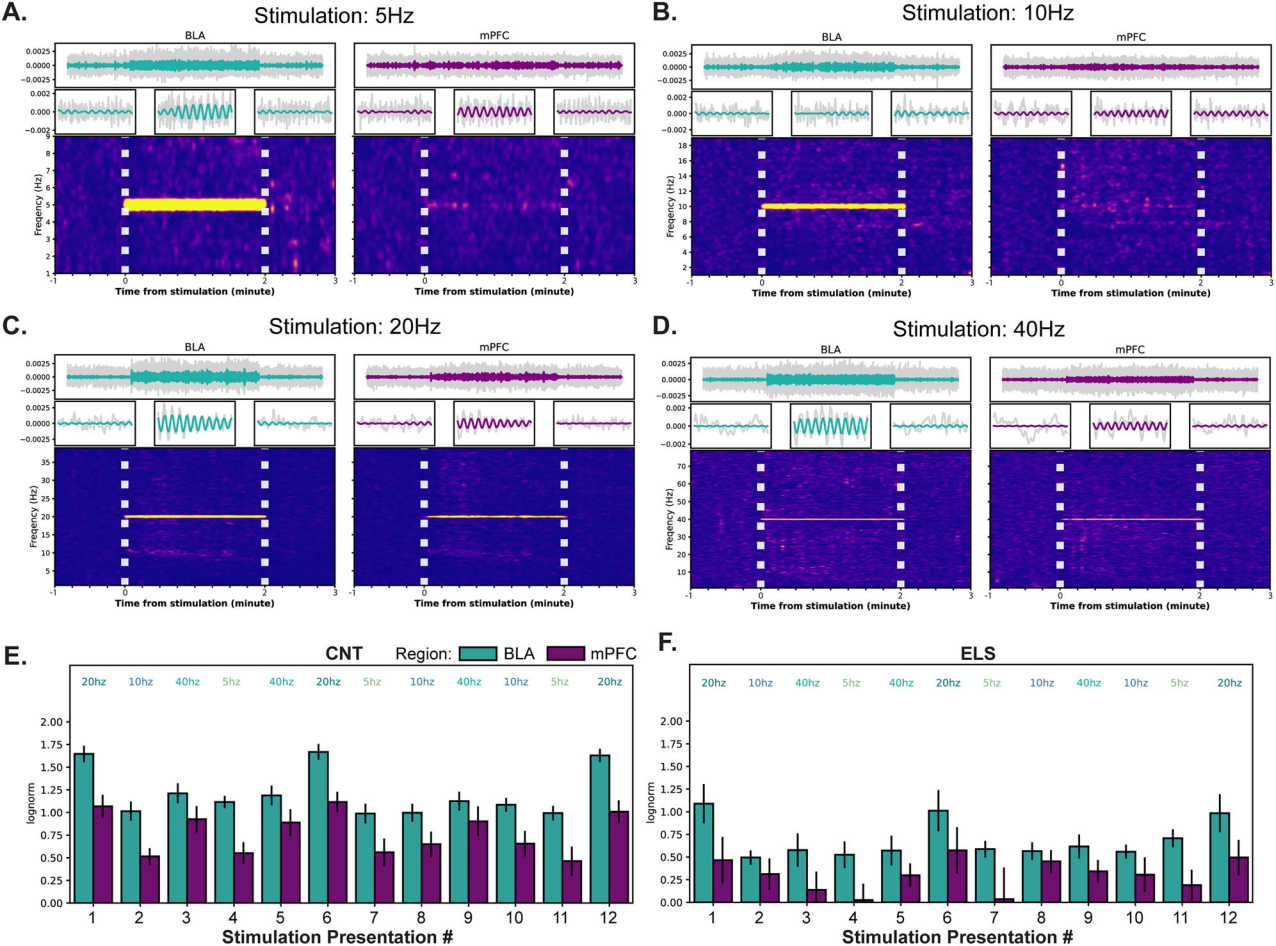
VTA_BLA_ robustly drives BLA and mPFC network activity. Representative traces and STFT transformations for a CNT animal during a (***A***) 5 Hz, (***B***) 10 Hz, (***C***) 20 Hz, and (***D***) 40 Hz trial. Top, Stimulated frequency LFP trace atop filtered (2–100 Hz) LFP traces (gray) from BLA (left) and mPFC (right). Middle, zoomed in portions from above traces show five periods before, during, and after 10 mW 640 nm photoexcitation. Bottom, STFT transformation from respective traces; horizontal white lines indicate onset/offset of 640 nm laser. ***E***, Grouped bar plot, organized by stimulation order, shows LFP power ratio between pulsed stimulation and baseline periods where power was measured at each stimulation frequency for CNT (blue, *n* = 7) animals. ***F***, Same as ***E*** with the exception that analyses performed on ELS (red, *n* = 8) animals. Spectrograms were not bandpass filtered but the power around stimulation frequencies were retrieved for the relative power plot in subpanels ***E*** and ***F***.

To determine whether dopaminergic inputs from the VTA had a mediating effect on BLA activity, we infused AAV-DIO-ChrR-tdT into VTA in standard-reared (CNT) DAT-Cre mice. Following a transfection period to allow for expression of ChrR-tdT in projection neurons from VTA, pulsed red light (640 nm) photoexcitation (5–40 Hz) in the BLA produced robust phase-locked LFP responses in the BLA (Fig. 1, Table 3) and coordinating BLA activity. It is important to note that repeated photostimulation did have any effect on power elevation (Fig. 2*E*,*F*; Table 4). These findings underscore the significant role that VTA DA has in regulating the BLA, supporting the validity of investigating how these dynamics might be altered under conditions of developmental stress.

**Table 3.** Statistical analyses for Figure 1

Description	Figure	Stat test	Stats report
DAT-Cre_BLA_5hz	Figure 1	One-sample *t* test, Bonferroni	*t* = 3.415, *p* = 0.005, *N* = 9
DAT-Cre_BLA_10hz	Figure 1	One-sample *t* test, Bonferroni	*t* = 3.088, *p* = 0.007, *N* = 9
DAT-Cre_BLA_20hz	Figure 1	One-sample *t* test, Bonferroni	*t* = 3.652, *p* = 0.003, *N* = 9
DAT-Cre_BLA_40hz	Figure 1	One-sample *t* test, Bonferroni	*t* = 2.970, *p* = 0.009, *N* = 9

Highlighted rows: *p* < 0.05.

**Table 4.** Statistical analyses for Figure 2

Description	Figure	Stat test	Stats report
CNT_BLA_5hz; trial*order	Figure 2*E*	Two-way RMANOVA	*F*_(2,12)_ = 0.132, *p* = 0.8776
CNT_BLA_10hz; trial*order	Figure 2*E*	Two-way RMANOVA	*F*_(2,12)_ = 0.055, *p* = 0.9467
CNT_BLA_20hz; trial*order	Figure 2*E*	Two-way RMANOVA	*F*_(2,12)_ = 0.296, *p* = 0.8096
CNT_BLA_40hz; trial*order	Figure 2*E*	Two-way RMANOVA	*F*_(2,12)_ = 0.34, *p* = 0.7187
CNT_mPFC_5hz; trial*order	Figure 2*E*	Two-way RMANOVA	*F*_(2,12)_ = 0.048, *p* = 0.9533
CNT_mPFC_1 0hz; trial*order	Figure 2*E*	Two-way RMANOVA	*F*_(2,12)_ = 0.242, *p* = 0.7891
CNT_mPFC_20hz; trial*order	Figure 2*E*	Two-way RMANOVA	*F*_(2,12)_ = 0.202, *p* = 0.8452
CNT_mPFC_40hz; trial*order	Figure 2*E*	Two-way RMANOVA	*F*_(2,12)_ = 0.092, *p* = 0.9123
ELS_BLA_5hz; trial*order	Figure 2*F*	Two-way RMANOVA	*F*_(2,14)_ = 1.426, *p* = 0.2731
ELS_BLA_10hz; trial*order	Figure 2*F*	Two-way RMANOVA	*F*_(2,14)_ = 0.546, *p* = 0.5909
ELS_BLA_20hz; trial*order	Figure 2*F*	Two-way RMANOVA	*F*_(2,14)_ = 0.471, *p* = 0.6355
ELS_BLA_40hz; trial*order	Figure 2*F*	Two-way RMANOVA	*F*_(2,14)_ = 0.044, *p* = 0.9575
ELS_mPFC_5hz; trial*order	Figure 2*F*	Two-way RMANOVA	*F*_(2,14)_ = 1.039, *p* = 0.3797
ELS_mPFC_10hz; trial*order	Figure 2*F*	Two-way RMANOVA	*F*_(2,14)_ = 0.808, *p* = 0.4656
ELS_mPFC_20hz; trial*order	Figure 2*F*	Two-way RMANOVA	*F*_(2,14)_ = 0.994, *p* = 0.3948
ELS_mPFC_40hz; trial*order	Figure 2*F*	Two-way RMANOVA	*F*_(2,14)_ = 0.08, *p* = 0.9234

**Figure 3.**
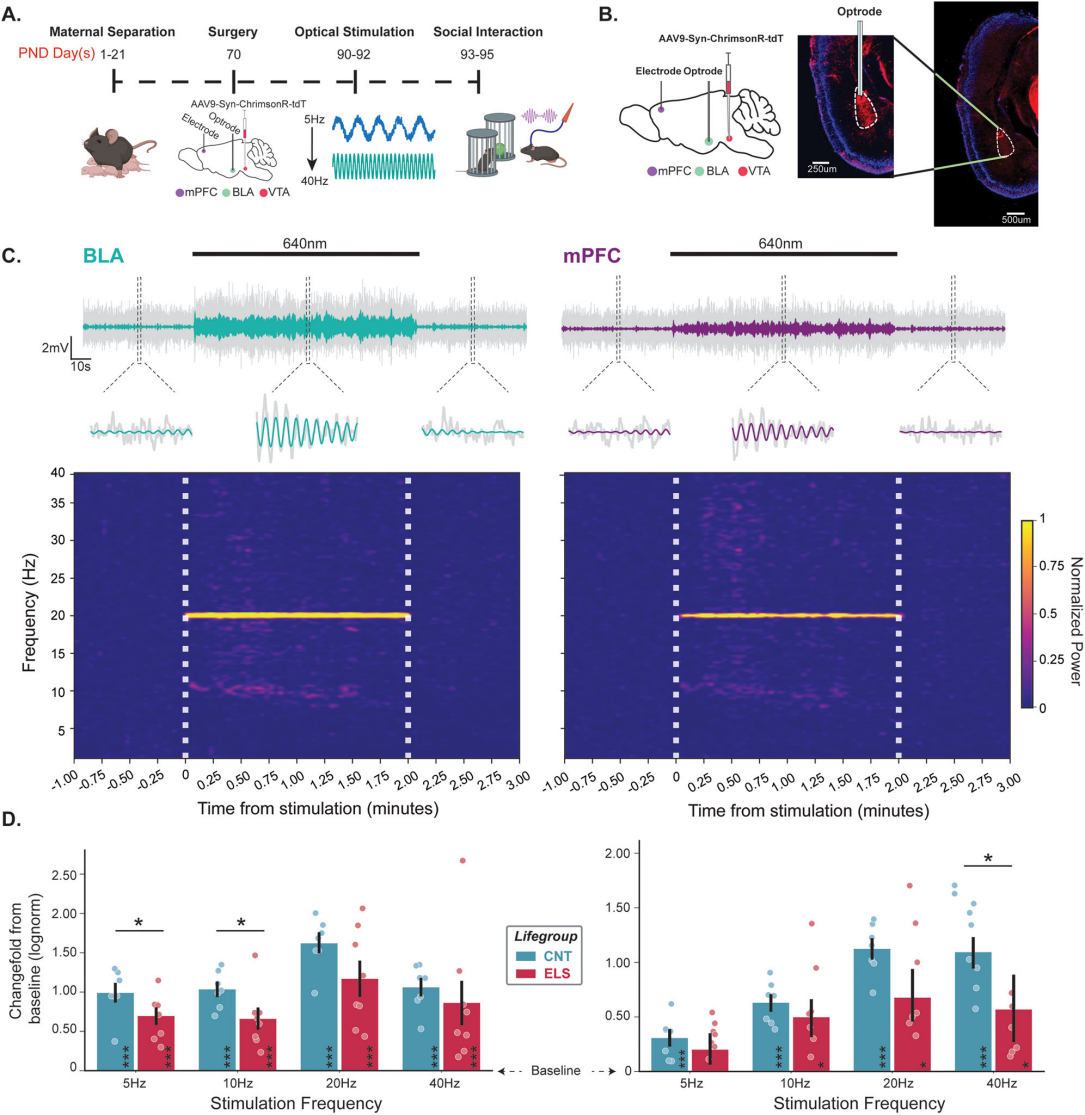
ELS inhibits ability for VTA_BLA_ to drive network activity. ***A***, Experimental timeline for ELS cohort. Timeline is identical for CNT cohort excluding maternal separation (D1–21). ***B***, Left, Schematic of sagittal view shows at P70, AAV-Syn-ChrRimsonR-tDT was injected into VTA, with an optrode and electrode placed into BLA and mPFC, respectively. Right, Representative fluorescence images of -tdT expression in the BLA. ***C***, Top, Representative 20 Hz LFP trace (colored) overlaid on raw LFP trace (2–100 Hz; gray) from BLA (left) and mPFC (right). Middle, Zoomed in portions from above traces show five periods before, during, and after 10 mW 640 nm photoexcitation. Bottom, STFT transformation from respective traces; vertical dashed white lines indicate onset/offset of 640 nm laser. Spectrogram was not bandpass filtered but the power around stimulation frequencies were retrieved for the relative power plot for ***D***. ***D***, Summary of LFP power ratio from BLA (left) and mPFC (right) between pulsed stimulation and baseline periods (baseline = 0) where power was measured at each stimulation frequency for CNT (blue, *n* = 7) and ELS (red, *n* = 8) conditions. Asterisks within bars indicate significant change from baseline. Asterisks atop horizontal bars indicate difference between CNT and ELS. *p < 0.05, **p < 0.01, ***p < 0.001. Error bars represent SEM.

To probe whether VTA→BLA (VTA_BLA_) activation influences BLA network activity, and if developmental stress alters these responses, we implemented an ELS protocol in male C57BL/6J mice, such that mice were either CNT or subjected to maternal separation stress for 3 h daily, 5 d per week from P1 to P21 (Fig. 3*A*). Following weaning and maturation, infusion of AAV-ChrR-tdT viral vector into VTA resulted in expression of ChrR-tdT in projection neurons from VTA, with strong terminal expression centered in BLA (Fig. 3*B*). The aforementioned photoexcitation experiment was repeated for these CNT and ELS animals, resulting in significant phase-locked LFP responses in both the BLA and mPFC, indicating that excitation of VTA inputs into the BLA is sufficient to control BLA network synchronization across a range of frequencies and entrain the mPFC network, with ELS attenuating this effect (Fig. 3*D*, Table 5). These results suggest that the ability for VTA inputs into the BLA to coordinate BLA→mPFC network states is impaired following ELS, which may give rise to aberrant processing of salient cues (Lauzon et al., 2009; Yizhar and Klavir, 2018) and, thus, maladaptive behaviors (Felix-Ortiz et al., 2016; for review see Likhtik and Paz, 2015).

**Table 5.** Statistical analyses for Figure 3

Description	Figure	Stat test	Stats report
CNT_BLA_5hz	Figure 3*D*, left	One-sample *t* test, Bonferroni	*t* = 16.481, *p* = <0.0001, *N* = 7
CNT_BLA_10hz	Figure 3*D*, left	One-sample *t* test, Bonferroni	*t* = 9.899, *p* = <0.0001, *N* = 7
CNT_BLA_20hz	Figure 3*D*, left	One-sample *t* test, Bonferroni	*t* = 22.555, *p* = <0.0001, *N* = 7
CNT_BLA_40hz	Figure 3*D*, left	One-sample *t* test, Bonferroni	*t* = 8.436, *p* = <0.0001, *N* = 7
CNT_mFPC_5hz	Figure 3*D*, right	One-sample *t* test, Bonferroni	*t* = 4.577, *p* = 0.0005, *N* = 7
CNT_mFPC_10hz	Figure 3*D*, right	One-sample *t* test, Bonferroni	*t* = 8.62, *p* = <0.0001, *N* = 7
CNT_mFPC_20hz	Figure 3*D*, right	One-sample *t* test, Bonferroni	*t* = 17.433, *p* = <0.0001, *N* = 7
CNT_mFPC_40hz	Figure 3*D*, right	One-sample *t* test, Bonferroni	*t* = 7.695, *p* = <0.0001, *N* = 7
ELS_BLA_5hz	Figure 3*D*, left	One-sample *t* test, Bonferroni	*t* = 7.193, *p* = <0.0001, *N* = 8
ELS_BLA_10hz	Figure 3*D*, left	One-sample *t* test, Bonferroni	*t* = 7.433, *p* = <0.0001, *N* = 8
ELS_BLA_20hz	Figure 3*D*, left	One-sample *t* test, Bonferroni	*t* = 5.127, *p* = 0.0001, *N* = 8
ELS_BLA_40hz	Figure 3*D*, left	One-sample *t* test, Bonferroni	*t* = 4.204, *p* = 0.0006, *N* = 8
ELS_mFPC_5hz	Figure 3*D*, right	One-sample *t* test, Bonferroni	*t* = 1.136, *p* = 0.139, *N* = 8
ELS_mFPC_10hz	Figure 3*D*, right	One-sample *t* test, Bonferroni	*t* = 2.641, *p* = 0.0108, *N* = 8
ELS_mFPC_20hz	Figure 3*D*, right	One-sample *t* test, Bonferroni	*t* = 2.456, *p* = 0.0151, *N* = 8
ELS_mFPC_40hz	Figure 3*D*, right	One-sample *t* test, Bonferroni	*t* = 2.192, *p* = 0.0244, *N* = 8
BLA; lifegroup*frequency	Figure 3*D*, left	Two-way mixed ANOVA	*F*_(3,39)_ = 0.538, *p* = 0.6591
mPFC; lifegroup*frequency	Figure 3*D*, right	Two-way mixed ANOVA	*F*_(3,39)_ = 1.889, *p* = 0.1474
BLA_5hz; lifegroup comparison	Figure 3*D*, left	Kruskal–Wallis	*H*_(1)_ = 4.339, *p* = 0.0372
BLA_10hz; lifegroup comparison	Figure 3*D*, left	Kruskal–Wallis	*H*(_1)_ = 4.835, *p* = 0.0279
BLA_20hz; lifegroup comparison	Figure 3*D*, left	Kruskal–Wallis	*H*_(1)_ = 1.929, *p* = 0.1649
BLA_40hz; lifegroup comparison	Figure 3*D*, left	Kruskal–Wallis	*H*_(1)_ = 2.625, *p* = 0.1052
mPFC_5hz; lifegroup comparison	Figure 3*D*, right	Kruskal–Wallis	*H*_(1)_ = 0.054, *p* = 0.817
mPFC_10hz; lifegroup comparison	Figure 3*D*, right	Kruskal–Wallis	*H*_(1)_ = 0.656, *p* = 0.4179
mPFC_20hz; lifegroup comparison	Figure 3*D*, right	Kruskal–Wallis	*H*_(1)_ = 2.263, *p* = 0.1325
mPFC_40hz; lifegroup comparison	Figure 3*D*, right	Kruskal–Wallis	*H*_(1)_ = 4.835, *p* = 0.0279

Highlighted rows: *p* < 0.05.

**Figure 4.**
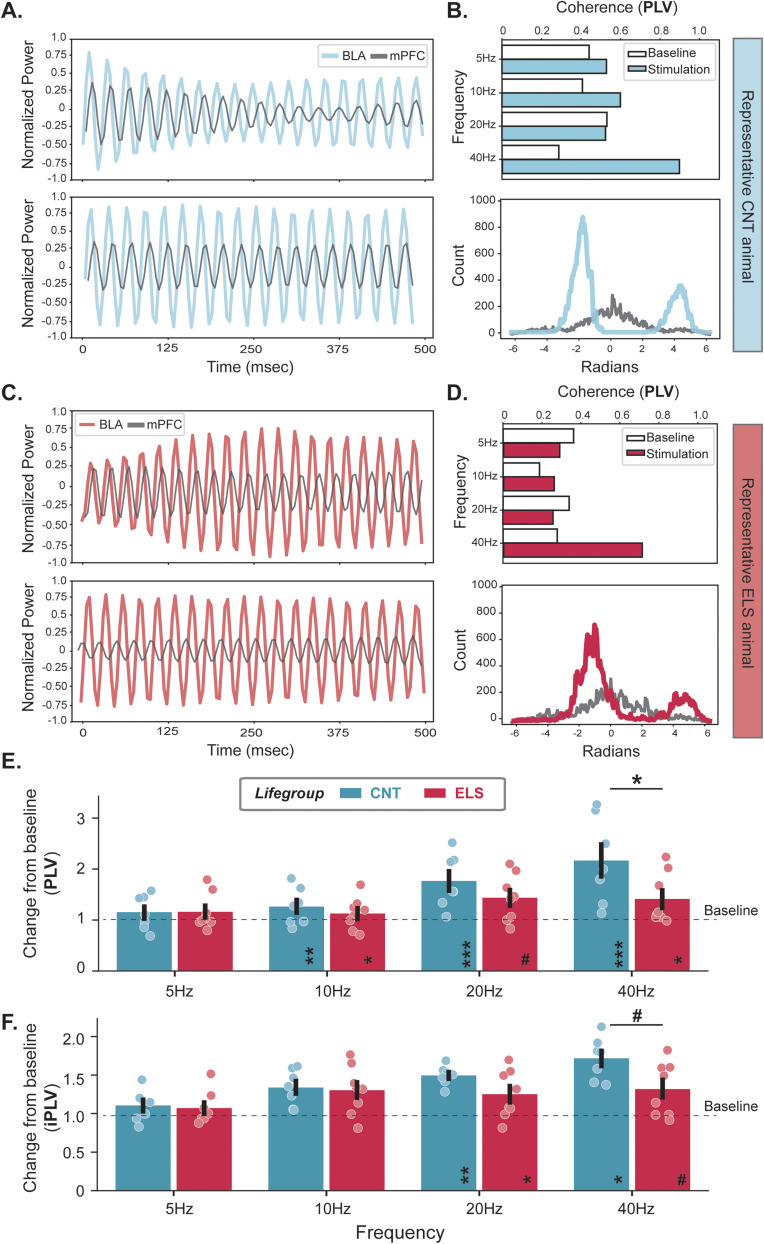
ELS minimizes VTA_BLA_→mPFC functional network connectivity. ***A***, Representative LFP traces from BLA and mPFC before (top) and during (bottom) 40 Hz VTA_BLA_ photoexcitation for a CNT mouse. ***B***, Top, Representative summary bar plots for animal in ***A*** detailing VTA-evoked BLA→mPFC coherence at each stimulation frequency. Bottom, Phase histogram for baseline (gray) and 40 Hz stimulation (red) in ***A***. ***C***, ***D***, Same analyses seen in ***A*** and ***B*** for a representative ELS mouse. Summary of LFP coherence (PLV, ***E***; and iPLV, ***F***) ratio between pulsed stimulation and baseline periods where BLA→mPFC coherence is measured at each stimulation frequency for CNT (blue, *n* = 7) and ELS (red, *n* = 8) conditions. Dashed horizontal line indicates baseline response. Asterisks within bars indicate significant change from baseline. Asterisks atop horizontal bars indicate difference between CNT and ELS. ^#^*p* ∼ 0.05, *p < 0.05, **p < 0.01, ***p < 0.001. Error bars represent SEM.

To examine the impact of ELS on VTA_BLA_-driven BLA→mPFC coordinated activity, we computed the PLV across a series of photoexcitation frequencies (Fig. 4*A–D*). As expected, photoactivation of VTA_BLA_ terminals promotes BLA activity to lead the activity in the mPFC (Fig. 4*B*,*D*; Table 5). Further, our results reveal an attenuation in the VTA-mediated coherence between BLA and mPFC following ELS (Fig. 4*E*). Specifically, VTA_BLA_ activation significantly enhances BLA→mPFC coherence in all frequencies >5 Hz, regardless of developmental stress. However, 40 Hz stimulation-induced phase coherence in the ELS group is significantly reduced relative to controls (Fig. 4*E*, Table 6)—a frequency previously shown to modulate sociability (i.e., interaction and avoidance behaviors) in rodents (Huang et al., 2020; Ferrara et al., 2023). The PLV calculation incorporates zero-phase differences that could arise from spurious synchrony such as volume conduction (Bruña et al., 2018). We therefore also computed the imaginary phase locking value (iPLV) that isolates noninstantaneous phase differences (Bruña et al., 2018). We find that VTA_BLA_-driven BLA→mPFC was significantly coordinated in CNT and ELS mice at 20 and 40 Hz (Fig. 4*F*, Table 6). Additionally, 40 Hz stimulation-induced phase coherence in ELS group was lower than CNT with a *p* = 0.051 (Fig. 4*F*, Table 6) in agreement with the PLV findings indicating that ELS reduces that VTA_BLA_-driven BLA→mPFC coordination.

**Table 6.** Statistical analyses for Figure 4

Description	Figure	Stat test	Stats report
CNT_5hz	Figure 4*E*	One-sample *t* test, Bonferroni	*t* = 1.376, *p* = 0.2179, *N* = 7
CNT_10hz	Figure 4*E*	One-sample *t* test, Bonferroni	*t* = 3.797, *p* = 0.0089, *N* = 7
CNT_20hz	Figure 4*E*	One-sample *t* test, Bonferroni	*t* = 10.459, *p* < 0.0001, *N* = 7
CNT_40hz	Figure 4*E*	One-sample *t* test, Bonferroni	*t* = 6.869, *p* = 0.0005, *N* = 7
ELS_5hz	Figure 4*E*	One-sample *t* test, Bonferroni	*t* = 0.953, *p* = 0.3723, *N* = 8
ELS_10hz	Figure 4*E*	One-sample *t* test, Bonferroni	*t* = 2.689, *p* = 0.0311, *N* = 8
ELS_20hz	Figure 4*E*	One-sample *t* test, Bonferroni	*t* = 2.292, *p* = 0.0556, *N* = 8
ELS_40hz	Figure 4*E*	One-sample *t* test, Bonferroni	*t* = 2.567, *p* = 0.0371, *N* = 8
BLA; lifegroup*frequency	Figure 4*E*	Two-way mixed ANOVA	*F*_(3,39)_ = 3.005, *p* = 0.0418
5hz; lifegroup comparison	Figure 4*E*	One-way ANOVA	*F*_(1,13)_ = 0.104, *p* = 0.7521
10hz; lifegroup comparison	Figure 4*E*	One-way ANOVA	*F*_(1,13)_ = 0.055, *p* = 0.8188
20hz; lifegroup comparison	Figure 4*E*	One-way ANOVA	*F*_(1,13)_ = 3.735, *p* = 0.0753
40hz; lifegroup comparison	Figure 4*E*	One-way ANOVA	*F*_(1,13)_ = 5.910, *p* = 0.0303
CNT_5hz	Figure 4*F*	One-sample *t* test, Bonferroni	*t* = 1.202, *p* = 0.2748, *N* = 7
CNT_10hz	Figure 4*F*	One-sample *t* test, Bonferroni	*t* = 1.900, *p* = 0.1062, *N* = 7
CNT_20hz	Figure 4*F*	One-sample *t* test, Bonferroni	*t* = 3.879, *p* = 0.0082, *N* = 7
CNT_40hz	Figure 4*F*	One-sample *t* test, Bonferroni	*t* = 3.665, *p* = 0.0105, *N* = 7
ELS_5hz	Figure 4*F*	One-sample *t* test, Bonferroni	*t* = 1.309, *p* = 0.2318, *N* = 8
ELS_10hz	Figure 4*F*	One-sample *t* test, Bonferroni	*t* = 1.148, *p* = 0.2886, *N* = 8
ELS_20hz	Figure 4*F*	One-sample *t* test, Bonferroni	*t* = 2.697, *p* = 0.0308, *N* = 8
ELS_40hz	Figure 4*F*	One-sample *t* test, Bonferroni	*t* = 2.351, *p* = 0.0509, *N* = 8
BLA; lifegroup*frequency	Figure 4*F*	Two-way mixed ANOVA	*F*_(3,39)_ = 1.682, *p* = 0.1865
5hz; lifegroup comparison	Figure 4*F*	One-way ANOVA	*F*_(1,13)_ = 0.002, *p* = 0.9665
10hz; lifegroup comparison	Figure 4*F*	One-way ANOVA	*F*_(1,13)_ = 0.592, *p* = 0.4554
20hz; lifegroup comparison	Figure 4*F*	One-way ANOVA	*F*_(1,13)_ = 1.702, *p* = 0.2146
40hz; lifegroup comparison	Figure 4*F*	One-way ANOVA	*F*_(1,13)_ = 4.619, *p* = 0.0510

Highlighted rows: *p* < 0.05.

**Figure 5.**
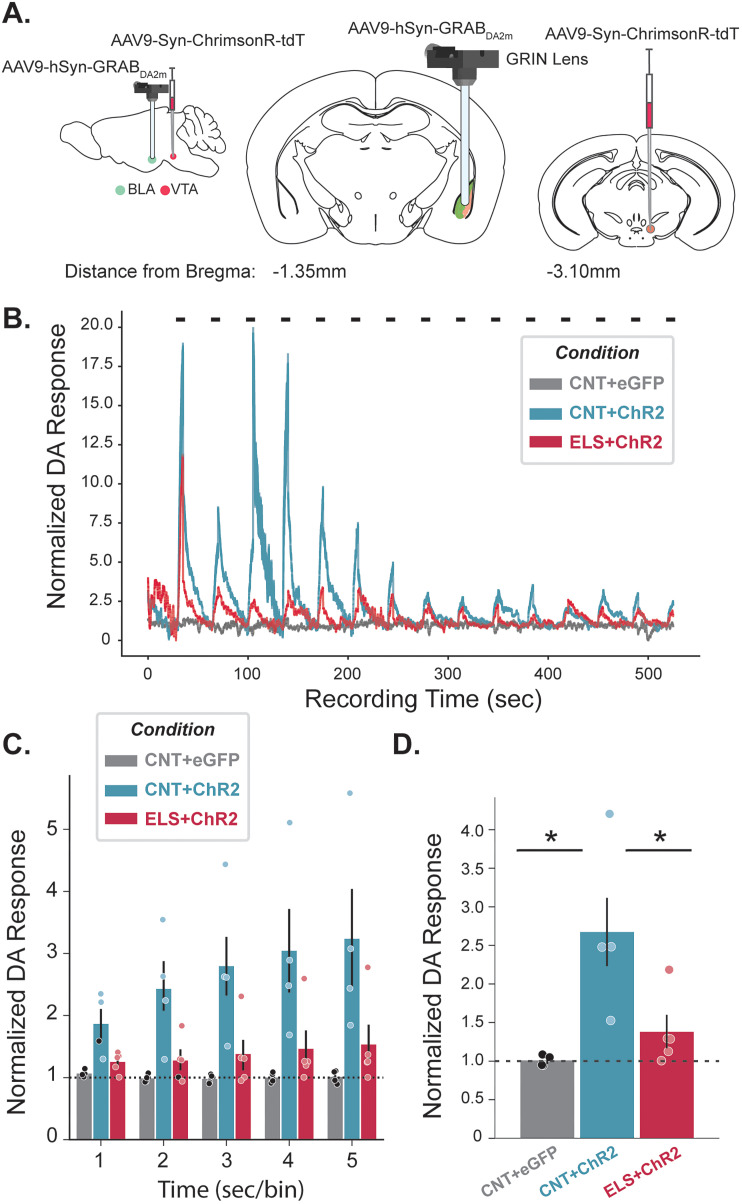
ELS reduces ELS reduces VTA_BLA_ DA response. ***A***, Schematic of sagittal and coronal views show AAV9-Syn-ChrRimsonR-tDT or AAV9-hSyn-eGFP was injected into VTA, AAV9-hSyn-GRAB_DA2m_ injected into BLA, with GRIN lens implanted above BLA. ***B***, Representative traces show dopamine (DA) response across a recording window consisting of 15–5 s trials of 20 mW red (620 ± 30 nm) 40 Hz photoexcitation indicated by horizontal black bars; eGFP, gray; CNT, blue; ELS, red. ***C***, Grouped bar plot shows trial-averaged normalized DA response for each second of 5 sec stimulation for eGFP (*n* = 4), CNT (*n* = 4), and ELS (*n* = 5) animals. Dashed horizontal line indicates baseline response. ***D***, Summary plot shows session-averaged normalized DA response for YFP, CNT, and ELS animals. *p < 0.05. Error bars represent SEM.

Building on the observed reduction in VTA_BLA_-mediated coherence between the BLA and mPFC following ELS, we hypothesized that ELS disrupts DA release from VTA_BLA_ projections, impairing DA-dependent modulation of network activity. To directly test this, we measured BLA DA responses by virally expressing a DA fluorescent sensor, GRABDA_2M_ (Beier et al., 2015), in the BLA of animals outfitted for microendoscopic imaging (Inscopix; Fig. 5*A*). Our initial inquiry evaluated the impact of ELS on the magnitude of DA response in the BLA following 40 Hz VTA_BLA_ activation, via photoexciting VTA ChrR expressing terminals in the BLA. We revealed that the VTA_BLA_-evoked DA response in CNT + ChrR animals were reliably greater than baseline fluorescence, reaching significance within 1 s of VTA_BLA_ terminal activation (Fig. 5*B,C*; Table 7). However, ELS robustly decreased this response, quantitatively resembling CNT + eGFP animals (Fig. 5*B–D*, Table 7). Collectively, these data demonstrate that ELS reduces VTA_BLA_ DA signaling.

**Table 7.** Statistical analyses for Figure 5

Description	Figure	Stat test	Stats report
DA (norm) response; group*bin	Figure 5*C*	Two-way ANOVA	*F*_(1,45)_ = 0.549, *p* = 0.8124
DA (norm) response; Control_eGFP*Control_ChrR	Figure 5*D*	Independent *t* test, Tukey	*t* = 3.292, *p* = 0.0229, N1 = 20; N2 = 25
DA (norm) response; Control_ChrR*ELS_ChrR_	Figure 5*D*	Independent *t* test, Tukey	*t* = 2.509, *p* = 0.0475, N1 = 25; N2 = 25
DA (norm) response; Control_eGFP*ELS_ChrR	Figure 5*D*	Independent *t* test, Tukey	*t* = −0.783, *p* = 0.7225, N1 = 20; N2 = 25

Highlighted rows: *p* < 0.05; N1, CNT; N2, ELS.

**Figure 6.**
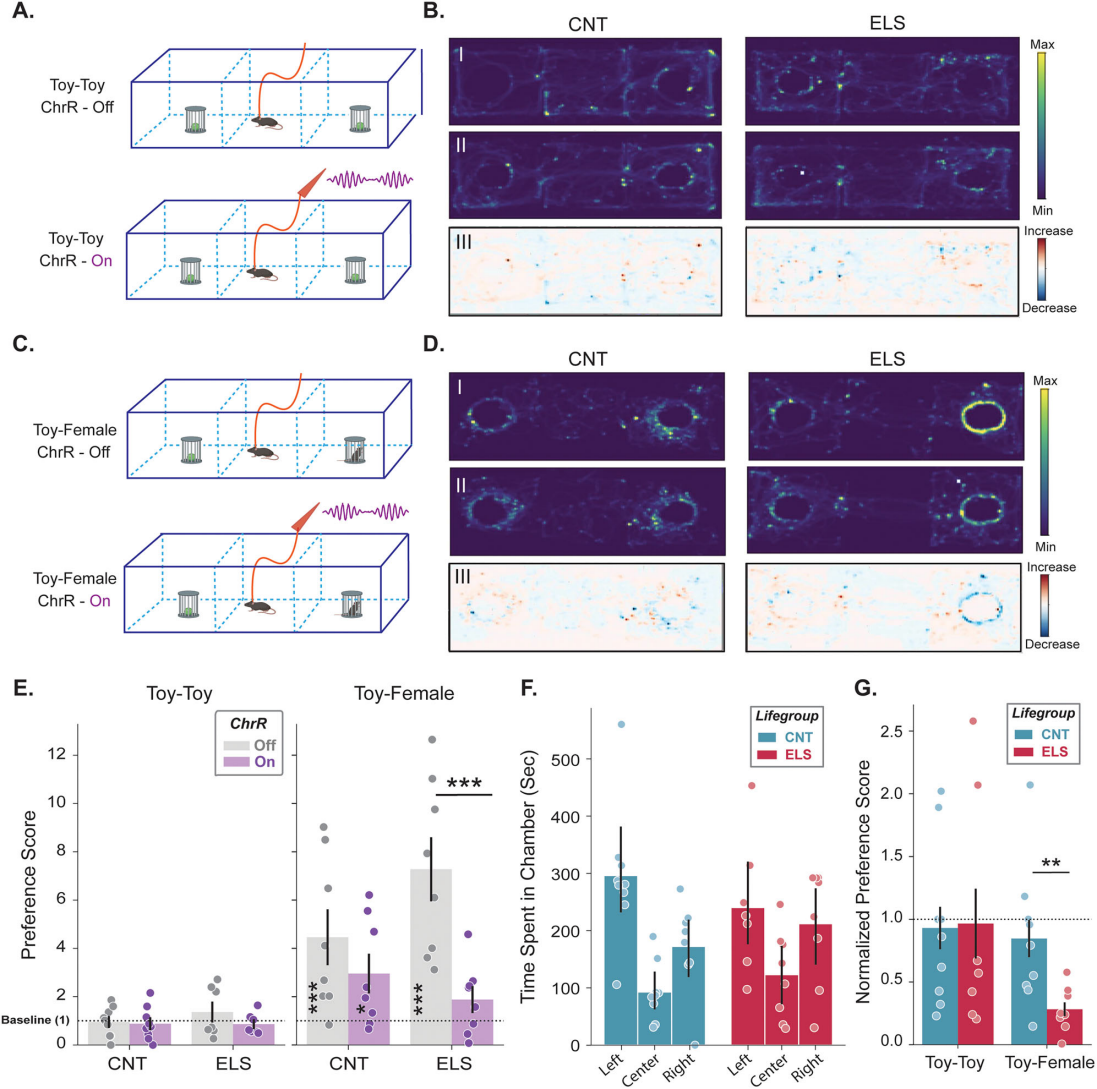
VTA_BLA_ activation in ELS reduces social interaction. ***A***, Schematic of three-chamber social interaction task (3CST) during Toy-Toy sessions for habituation (ChrR-Off, top) and testing (ChrR-On, bottom) trials. ***B***, Representative heatmaps during 3CST show location for a CNT (left) and ELS (right) animal during habituation and testing trials, BI and BII, respectively. Heat, Min-Max scaled duration across trial. BIII shows subtraction plots, with heat indicating the difference in time spent at each location between testing and habituation trials. ***C***, ***D***, Same as ***A*** and ***B*** except interaction objects are a toy and novel, female conspecific (Toy-Female). ***E***, Grouped bar plots show interaction preference scores for CNT (*n* = 7) and ELS (*n* = 8) animals across habituation and testing trials for Toy-Toy (left) and Toy-Female (right) sessions; value of 1 is equivalent time spent in stimulation and opposite side. Asterisks centered over trial: one side of chamber is significantly preferred. Asterisks centered across trials: preference scores during testing trials significantly differed from habituation trials. ***F***, Grouped bar plot shows the effect of ELS on locomotion activity in 3CST, displayed as time (seconds) spent in each chamber during each animal (control, *N* = 7; ELS, *N* = 8) habituation trial. Mixed ANOVA (position × lifegroup) excluding center position revealed no significant effect (*p* > 0.05). ***G***, Grouped bar plot shows normalized preference scores from ***E*** for CNT and ELS animals across Toy-Toy and Toy-Female sessions; value of 1: equivalent preference score between habituation and testing trials. Preference score is the percentage of time the mouse spends in the stimulation zone versus both sides of the area; a value >1 suggests a preference for the stimulation side. Normalized preference score compares the PS during testing with the habituation trial; a value >1 indicates increased stimulation-side preference compared with preference during habituation. *p < 0.05, **p < 0.01, ***p < 0.001. Error bars represent SEM.

Noting the impact of ELS on VTA_BLA_ DA signaling, we focused our attention on interrogating the impact of ELS on innate motivated behaviors, such as social interaction. We assessed the impact of ELS on social preference using a 3CST which evaluated an animal's proclivity to investigate either an inanimate object (fake mouse toy) or novel, female conspecific (female) with or without rhythmic VTA_BLA_ activation (Fig. 6*A–D*). Then, 40 Hz stimulation was chosen for terminal activation because BLA gamma (30–80 Hz) is known to be crucial for emotional/social processing (Stujenske et al., 2014; Felix-Ortiz et al., 2016), and it is the frequency band where rearing conditions caused a significant difference in BLA→mPFC functional connectivity (Fig. 4*E*,*F*). CNT and ELS animals showed comparable interaction levels when presented with toys, with no effect of VTA_BLA_ photoexcitation (On) on interaction preference in comparison with trials in the absence of photoexcitation (Off; Figs. 6*E*, left; Table 8). Further scrutiny regarding whether ELS impacted the capability of mice to interact, specifically looking at locomotor activity in terms of time spent within each 3CST chamber, revealed that ELS animals had similar locomotor behavior as CNT animals (Fig. 6*F*, Table 8). Conversely, when presented with the decision to interact with a toy or a female in the absence of VTA_BLA_ activation, animals robustly preferred to socialize (Fig. 6*E*, right; Table 8). However, in trials where VTA_BLA_ activation occurred, ELS animals changed their preference, erring on the side of social avoidance, a behavior that was not exhibited in CNT animals with intact VTA_BLA_ DA signaling (Fig. 6*G*, Table 8). These data suggest that the changes in VTA_BLA_ DA signaling impair social interaction.

**Table 8.** Statistical analyses for Figure 6

Description	Figure	Stat test	Stats report
CNT_off_toy_toy	Figure 6*E*, left	One-sample *t* test, Bonferroni	*t* = −0.615, *p* = 0.7264, *N* = 8
CNT_on_toy_toy	Figure 6*E*, left	One-sample *t* test, Bonferroni	*t* = −1.395, *p* = 0.9067, *N* = 8
ELS_off_toy_toy	Figure 6*E*, left	One-sample *t* test, Bonferroni	*t* = 0.69, *p* = 0.2508, *N* = 7
ELS_on_toy_toy	Figure 6*E*, left	One-sample *t* test, Bonferroni	*t* = −0.004, *p* = 0.5017, *N* = 7
CNT_off_toy_female	Figure 6*E*, right	One-sample *t* test, Bonferroni	*t* = 3.852, *p* = 0.0008, *N* = 8
CNT_on_toy_female	Figure 6*E*, right	One-sample *t* test, Bonferroni	*t* = 2.32, *p* = 0.0213, *N* = 8
ELS_off_toy_female	Figure 6*E*, right	One-sample *t* test, Bonferroni	*t* = 7.668, *p* = <0.0001, *N* = 7
ELS_on_toy_female	Figure 6*E*, right	One-sample *t* test, Bonferroni	*t* = 1.035, *p* = 0.1627, *N* = 7
Toy-Toy; lifegroup*ChrR	Figure 6*E*, left	Two-way mixed ANOVA	*F*_(1,13)_ = 0.787, *p* = 0.391
Toy-Female; lifegroup*ChrR	Figure 6*E*, right	Two-way mixed ANOVA	*F*_(1,14)_ = 5.155, *p* = 0.0395
Locomotion; position*lifegroup	Figure 6*F*	Two-way mixed ANOVA	*F*_(1,14)_ = 1.039, *p* = 0.3240
Toy-Toy; lifegroup comparison	Figure 6*G*	Independent *t* test	*t* = 1.187, *p* = 0.2583, N1 = 8; N2 = 7
Toy-Female; lifegroup comparison	Figure 6*G*	Independent *t* test	*t* = 3.905, *p* = 0.0016, N1 = 8; N2 = 7

Highlighted rows: *p* < 0.05; N1, CNT; N2, ELS.

**Figure 7.**
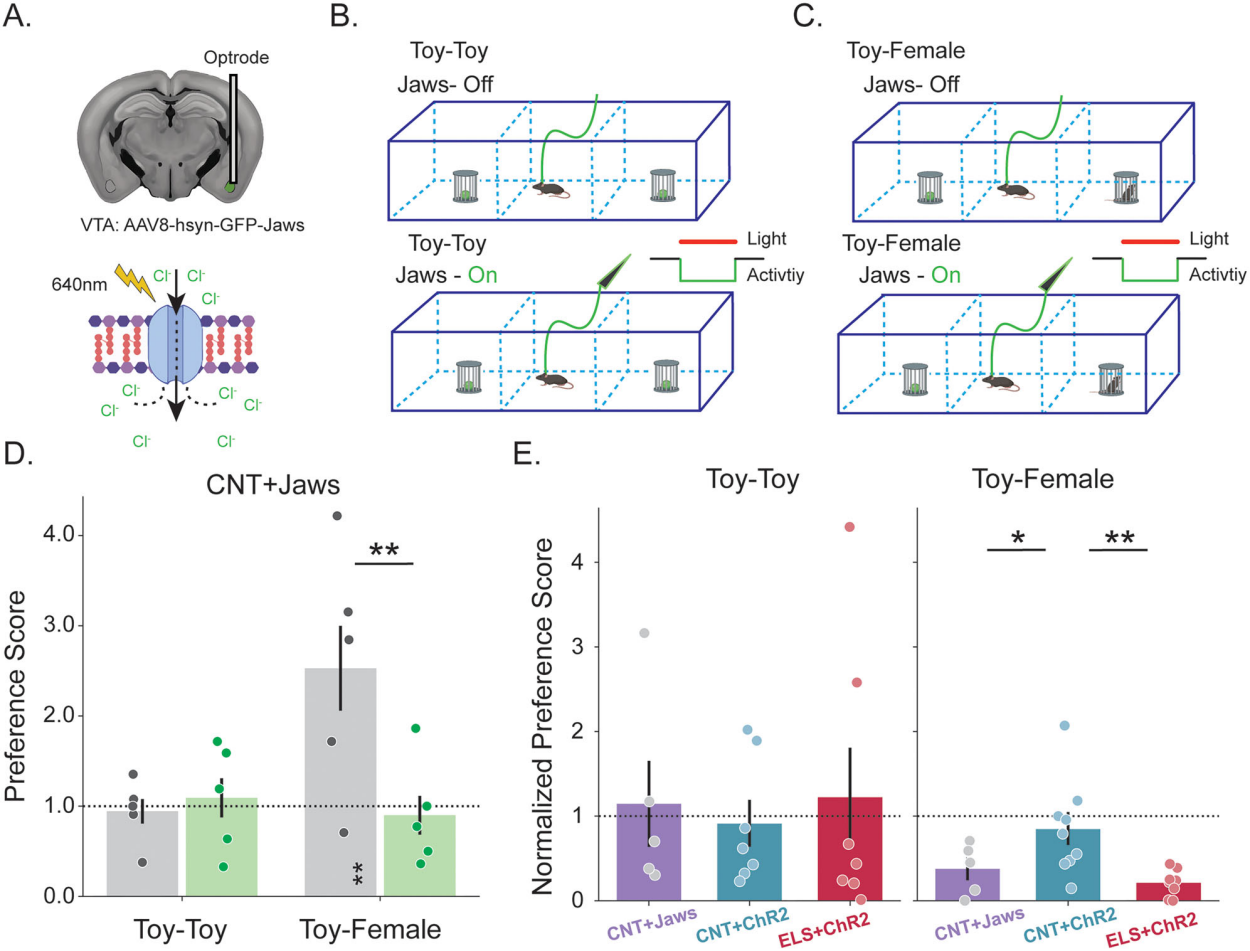
VTA_BLA_ inhibition mimics social deficit observed following ELS + VTA_BLA_ excitation. ***A***, Top, Schematic of coronal slice showing AAV8-hsyn-GFP-Jaws was injected into VTA with optrode implanted into BLA. Bottom, Schematic of inhibitory mechanism of red-shifted Jaws acting on anion-gated membrane following 640 nm photostimulation. ***B***, Schematic of 3CST during Toy-Toy sessions for habituation (Jaws-Off, Top) and testing (Jaws-On, Bottom) trials. ***C***, Schematic showing same experimental set-up as in ***B*** with the exception being interaction objects are a toy and novel, female conspecific (Toy-Female). ***D***, Grouped bar plots show interaction preference scores for Toy-Toy and Toy-Female sessions habituation and testing; value of 1 is equivalent time spent in stimulation and opposite side. Significance stars centered over trial: one side of chamber is significantly preferred. Significance stars centered across trials: preference scores during testing trials significantly differed from habituation trials. ***E***, Grouped bar plot shows normalized preference scores from ***D*** for CNT + Jaws animals (*n* = 5) as well as CNT + ChrR and ELS + ChrR animals (from Fig. 6) across Toy-Toy and Toy-Female sessions; value of 1: equivalent preference score between habituation and testing trials. Preference score is the percentage of time the mouse spends in the stimulation zone versus both sides of the area; a value >1 suggests a preference for the stimulation side. Normalized preference score compares the PS during testing with the habituation trial; a value >1 indicates increased stimulation-side preference compared with preference during habituation. *p < 0.05. **p < 0.01.

To directly test whether impaired functional connectivity between the VTA and BLA following ELS contributes to altered social interaction, we silenced VTA_BLA_ signaling and examined the impact on social preference. In an adaptation of the experimental protocol detailed in Figure 2*A*, we infused a red-light–sensitive chloride pump-encoding viral vector (AAV-GFP-JAWS; Jaws) into VTA to promote VTA_BLA_ terminus expression aimed at inhibiting VTA-mediated activity (Fig. 7*A*). Given that we saw an ELS-specific reduction in VTA_BLA_ signaling, we posited that inhibiting VTA_BLA_ information in CNT animals would result in a similar social avoidance behavior exhibited by ELS animals. CNT + Jaws mice were then subjected to the same 3CST detailed in Figure 6, with the exception that during testing trials, VTA_BLA_ signaling was inhibited (Fig. 7*B–C*). Interaction levels were comparable between habituation and testing trials when CNT + Jaws mice were presented with toys. Meanwhile, when presented with a female conspecific, these animals significantly increased their social interaction, a preference that was significantly decreased during VTA_BLA_ inhibition (Fig. 7*D*, Table 9). When we directly compare the results between the VTA_BLA_ excitation and inhibition experiments, our hypothesis is borne out: VTA_BLA_ inhibition causes a reduction in socializing in CNT animals, a VTA_BLA_-induced phenotype exhibited following ELS, which is not observed in VTA_BLA_-stimulated CNT animals (Fig. 7*E*).

**Table 9.** Statistical analyses for Figure 7

Description	Figure	Stat test	Stats report
Off_toy_toy	Figure 7*D*	One-sample *t* test, Bonferroni	*t* = 0.56, *p* = 0.2953, *N* = 10
On_toy_toy	Figure 7*D*	One-sample *t* test, Bonferroni	*t* = −0.554, *p* = 0.7027, *N* = 10
Off_toy_female	Figure 7*D*	One-sample *t* test, Bonferroni	*t* = 3.333, *p* = 0.0052, *N* = 10
On_toy_female	Figure 7*D*	One-sample *t* test, Bonferroni	*t* = −0.244, *p* = 0.5934, *N* = 10
Toy_toy; Jaws comparison	Figure 7*D*	Paired *t* test, one-tailed	*t* = 1.052, *p* = 0.176, *N* = 5
Toy_female; Jaws comparison	Figure 7*D*	Paired *t* test, one-tailed	*t* = 3.315, *p* = 0.0108, *N* = 5
Toy-Toy; group*stim	Figure 7*E*, left	Two-way mixed ANOVA	*F*_(2,15)_ = 0.481, *p* = 0.6273
Toy-Female; group*stim	Figure 7*E*, right	Two-way mixed ANOVA	*F*_(2,16)_ = 3.986, *p* = 0.0394
CNT_Jaws_toy_toy	Figure 7*F*, left	One-sample *t* test, Bonferroni	*t* = −0.483, *p* = 0.6781, *N* = 5
CNT_ChrR_toy_toy	Figure 7*F*, left	One-sample *t* test, Bonferroni	*t* = 0.845, *p* = 0.2056, *N* = 7
ELS_ChrR_toy_toy	Figure 7*F*, left	One-sample *t* test, Bonferroni	*t* = 1.187, *p* = 0.1292, *N* = 8
CNT_Jaws_toy_female	Figure 7*F*, right	One-sample *t* test, Bonferroni	*t* = 2.382, *p* = 0.0269, *N* = 5
CNT_ChrR_toy_female	Figure 7*F*, right	One-sample *t* test, Bonferroni	*t* = 1.105, *p* = 0.1439, *N* = 7
ELS_ChrR_toy_female	Figure 7*F*, right	One-sample *t* test, Bonferroni	*t* = 4.311, *p* = 0.0012, *N* = 8
Toy_toy; CNT_ ChrR*CNT_Jaws	Figure 7*F*, left	Independent *t* test	*t* = −0.392, *p* = 0.6459, N1 = 7, N2 = 5
Toy_toy; CNT_ ChrR*ELS_ChrR	Figure 7*F*, left	Independent *t* test	*t* = −0.456, *p* = 0.6717, N1 = 7, N2 = 8
Toy_toy; CNT_Jaws*ELS_ChrR	Figure 7*F*, left	Independent *t* test	*t* = −0.096, *p* = 0.5373, N1 = 5, N2 = 8
Toy_female; CNT_ ChrR*CNT_Jaws	Figure 7*F*, right	Independent *t* test	*t* = 1.454, *p* = 0.0384, N1 = 7, N2 = 5
Toy_female; CNT_ ChrR*ELS_ChrR	Figure 7*F*, right	Independent *t* test	*t* = 2.684, *p* = 0.0104, N1 = 7, N2 = 8
Toy_female; CNT_Jaws*ELS_ChrR	Figure 7*F*, right	Independent *t* test	*t* = 1.669, *p* = 0.0839, N1 = 5, N2 = 8

Highlighted rows: *p* < 0.05; N1, CNT; N2, ELS.

## Discussion

The results of this study advance our understanding of the mechanisms through which ELS affects brain circuitry and behaviors in mice. Our experiments with control DAT-Cre animals initially demonstrated that DA release from the VTA effectively drives synchronized BLA activity, establishing a baseline for neural dynamics under normal conditions. Subsequent investigations highlight the importance of VTA_BLA_ signaling in coordinating BLA→mPFC activity and influencing social preference, with ELS disrupting this coordinated signaling associated with altered social behavior. Specifically, we observed that rhythmic activation of VTA_BLA_ terminals entrained BLA network activity, elevated BLA→mPFC coherence, and increased BLA dopamine levels in control mice. In contrast, following ELS, we observed weakened VTA-mediated BLA and mPFC entrainment, diminished BLA→mPFC coherence, and reduced VTA_BLA_ DA levels. Interestingly, optogenetic inhibition of VTA_BLA_ signaling in ELS mice induced social avoidance. Mimicking the impaired VTA_BLA_ signaling observed in ELS mice by optogenetic inhibition of VTA_BLA_ activity in control mice was sufficient to impair social interaction. Given the pervasiveness of ACEs and their well-established comorbidity with psychiatric disorders (Humphreys et al., 2020; Sheridan and McLaughlin, 2020; Berman et al., 2022), the elucidation of the mechanism behind such vulnerabilities remains paramount. Our study highlights the pivotal role of the MCL system, more specifically the interaction between VTA and BLA, in mediating these behavioral outcomes.

The robust modulation of BLA activity by VTA DA in control DAT-Cre animals, as detailed in our initial experiments, establishes a clear dopamine-derived control of this crucial neural circuit under normal conditions.

While it is possible that other neurotransmitters such as glutamate and GABA may influence the mediating role of VTA_BLA_ signaling, the VTA is predominantly composed of DA neurons, which are well known to govern reward valuation. The anatomical and functional connections between the VTA and the BLA have been extensively documented to play a significant role in reward processing and motivational behaviors (Hou et al., 2024). Furthermore, developmental stress is known to critically shape early neural reward circuitry, affecting how these systems respond to stressors later in life (Lowes et al., 2021; Smith and Torregrossa, 2021; Sun et al., 2021; Hou et al., 2024). This backdrop, in conjunction with our foundational results, motivated our inquiry into how such developmental challenges could alter these dopaminergic dynamics and subsequent behavioral outcomes.

Maternal neglect has been robustly associated with cognitive impairments and increased susceptibility to psychiatric disorders (Delavari et al., 2016; Bolton et al., 2018; Reshetnikov et al., 2020). Our results support this by demonstrating that ELS can modulate the neurophysiological pathways implicated in several stress-linked behaviors impacting social reward. However, while the involvement of the MCL system (particularly the MCL DA system) in stress vulnerability has been documented (Fibiger and Phillips, 1988; Sullivan and Dufresne, 2006; Butts et al., 2011), our study provides a breakthrough by elucidating the impact of VTA on BLA and mPFC network dynamics, bringing to light how ELS affects their functional connectivity which is known to influence behavioral states. These results reveal that ELS affects the quantity of BLA-targeted VTA DA neurons and thus alters DA release, which could impair BLA→mPFC information flow. Given that reduced DA tone in the prefrontal cortex (PFC) has been associated with impaired cognitive function and increased susceptibility to stress (for a review see Arnsten, 2009), and that altered connectivity between the PFC and the amygdala leads to heightened emotional reactivity and impaired regulation (Tottenham, 2012), we propose that ELS alters social behaviors by impacting DA circuitry through the BLA. Our findings underscore the critical interplay between these regions, especially in the context of ELS; disrupting the BLA→mPFC network has profound behavioral consequences, notably social avoidance, thereby offering potential pathways for therapeutic interventions.

Specifically, the Jaws-mediated silencing experiment (Fig. 7) inhibiting VTA→BLA projections in control mice using Jaws led to social avoidance behavior, reinforcing the notion that this pathway is essential for assigning positive salience to social stimuli under physiological conditions. These results align well with previous literature indicating that dopaminergic transmission from the VTA is necessary for social motivation and approach behavior (Tye et al., 2013; Gunaydin et al., 2014). Interestingly, our study reveals that activating the same pathway in ELS mice not only fails to enhance social interaction but actively disrupts it. This paradoxical effect may be attributed to the altered neurophysiological landscape shaped by ELS, which compromises VTA→BLA coherence and dopaminergic tone, ultimately impacting how these circuits process social information.

Studies have also shown that ELS-induced anhedonia (loss of pleasure capacity) has been linked to aberrant signaling interactions across the MCL circuit (Chocyk et al., 2011; for review see Bolton et al., 2017). In response to these disruptions, ELS animals likely develop compensatory mechanisms, such as strengthening synaptic connections (e.g., synaptic efficacy), remapping neural pathways, or adjusting excitatory/inhibitory signaling to maintain homeostasis. These adaptive changes could explain why ELS mice exhibit relatively normal social behavior under baseline conditions. However, when the MCL network is challenged—either through VTA_BLA_ excitation or inhibition—these compensatory circuits may become destabilized, resulting in altered behavioral outcomes. Specifically, the activation of VTA→BLA projections in ELS mice might disrupt these adapted pathways, impairing the processing of social cues and causing social avoidance behaviors

This framework aligns with the observation that baseline social behavior in ELS mice appears unaffected due to the recruitment of alternative neural mechanisms. Evidence from the literature supports this compensatory adaptation hypothesis, as chronic stress and developmental adversity can drive plasticity at the circuit level, allowing engagement of alternative pathways to preserve behavioral function (McEwen and Morrison, 2013; Johnson and Young, 2015). Therefore, the aberrant activation of the VTA→BLA pathway in ELS mice might interfere with these circuits, leading to maladaptive social behaviors. Our data suggest that the reduced coherence between BLA and mPFC does not merely reflect a “negative” signal but rather signifies an impaired capacity to transmit a coherent and contextually appropriate “positive” salience signal. This disruption compromises the delicate balance needed for adaptive social interactions, explaining the differential impact of VTA→BLA activation in control versus ELS animals.

These mechanisms together, or in and of themselves, could lead to differential behavioral outcomes, particularly when this MCL network is challenged (e.g., VTA_BLA_ excitation or inhibition). Accordingly, we propose that the MCL system is targeted during ELS and that the observed alteration in social motivation is substantiated in VTA DA driving (or failing to drive) oscillations across the BLA and mPFC which govern interaction behaviors. Future work should focus on identifying these compensatory pathways and elucidating how ELS rewires mesocorticolimbic circuitry to support social behaviors. Additionally, assessing whether pharmacological or neuromodulatory interventions can restore VTA→BLA coherence and improve social behavior in ELS mice is crucial for developing therapeutic strategies for stress-induced social deficits. While further research is undeniably required, especially in understanding the broader implications of these findings, our study constitutes a significant step in understanding the neural underpinnings of stress vulnerability.

## Data Availability

Further data supporting the findings are available upon reasonable request to the corresponding author.
